# The Percentage of Fat in Infants' Food

**Published:** 1900-04-28

**Authors:** 


### THE PERCENTAGE OF FAT IN INFANTS' FOOD.

Messrs. Gilbert, Kimpton, and Co. write:—On March 3rd last you kindly gave "Milkine" Food and Tablets a notice, but you criticised it as being deficient in fat. We have communicated with our friends the manufacturers, and have just received their reply as follows:—

"In regard to the question of fat, the practical results that have come from Milkine show that it is rich enough in fats for any baby. The question is not often discussed by the medical profession here, but some of them advise us that they use a very small quantity of cream in mixing; we hold, however, that this is very unnecessary, and, as we said above, the practical use shows that we are right. The analysis of mothers' milk shows the fat as varying in the milk from different women from 1½ to even 2 per cent. Some foods that we know put out an analysis showing a great deal of fat, but we doubt if their food, mixed according to directions, will give the same analysis as they claim, and if it did, there would be entirely too much in the food to be suitable for a baby."

We trust that you will be so good as to amend the notice, and thanking you for kindly giving this matter your kind attention.

⁂ In reference to the above, our examiner of proprietary foods writes:—"I beg to return Messrs. Gilbert and Kimpton's letter *re* 'Milkine.' I do not deny that human milk sometimes possesses no higher percentage of fats than 1·5 or 2 per cent.; but when such is the case it is condemned by all authorities as a food for infants. Average human milk contains 4 per cent. fat, and this is accepted as the normal standard for infantile requirements. Artificial foods which contain a lower percentage cannot be regarded as physiologically sufficient foods. Reference to the three leading authorities on infant feeding will fully confirm the criticism that 'Milkine' is not a complete food for infants. *See* 'Diseases of Infancy and Childhood,' Holt, pages 171, 130, 157. 'Clinical Examination and Treatment of Children,' Thomson, page 213. 'Artificial Feeding of Infants,' Cheadle, page 108 et seq. I do not think any alteration or modification is called for in my notice *re* 'Milkine.' It is a fair and unprejudiced criticism. If 'Milkine' errs in respect of deficiency in fat it is no greater sinner than any other proprietary food."

